# Graphene quantum dots remodel osteogenesis of human periodontal ligament stem cells under inflammatory microenvironment: an *in vitro* and *in vivo* study

**DOI:** 10.3389/fbioe.2026.1879913

**Published:** 2026-08-26

**Authors:** Sicheng Deng, Xiaona Wu, Shaoyong Chen, Yulu Xie, Lingsan Ran, Haiyan Xue, Qiuling Pang, Kuangdi Xin, Yuehua You, Rongmin Qiu

**Affiliations:** 1 College and Hospital of Stomatology, Guangxi Medical University, Guangxi Key Laboratory of Oral and Maxillofacial Rehabilitation and Reconstruction, Guangxi Clinical Research Center for Craniofacial Deformity, Guangxi Key Laboratory of Oral and Maxillofacial Surgery Disease Treatment, Guangxi Health Commission Key Laboratory of Prevention and Treatment for Oral Infectious Diseases, Key Laboratory of Research and Application of Stomatological Equipment, Education Department of Guangxi Zhuang Autonomous Region, Nanning, Guangxi, China; 2 Key Laboratory of Longhua, Department of Stomatology, Longhua People’s Hospital, Shenzhen, China; 3 School of Stomatology, Southern Medical University, Guangzhou, China

**Keywords:** alveolar bone regeneration, graphene quantum dots, human periodontal ligament stem cell, migration, osteogenic differentiation, periodontitis, proliferation

## Abstract

**Objective:**

This study aims to provide experimental basis that underpins the potential application of graphene quantum dots (GQDs) as a therapeutic strategy for periodontitis.

**Methods and Materials:**

An inflammatory circumstance was established by Porphyromonas gingivalis lipopolysaccharide (P.g-LPS). The effects of GQDs on human periodontal ligament stem cells (hPDLSCs) proliferation, migration and osteogenic differentiation *in vitro* were assessed using Cell Counting Kit-8 (CCK-8), Transwell assay, alkaline phosphatase (ALP) and alizarin red S (ARS) staining, ALP activity and ARS semi-quantification. The anti-inflammatory activity was evaluated by Enzyme-linked immunosorbent assay (Elisa). The underlying osteogenic mechanism was initially explored by mRNA sequencing (mRNA-Seq) and further correlated with gene and protein expression changes via quantitative real-time PCR (RT-qPCR) and Western blot (WB) analysis. Furthermore, the *in vivo* efficacy and biosafety of GQDs were evaluated in a rat periodontitis model using micro computed tomography (Micro-CT) and histomorphometric analysis.

**Result:**

*In vitro*, GQDs exhibited favorable cytocompatibility and enhanced the viability, migration, osteogenesis of hPDLSCs under normal conditions. In inflammatory microenvironment, GQDs significantly counteracted the suppression of P.g-LPS on hPDLSCs proliferation, migration, osteogenic differentiation, and notably reduced the levels of tumor necrosis factor-α (TNF-α), interleukin-1β (IL-1β) and interleukin-6 (IL-6). Mechanistically, mRNA-Seq identified the phosphoinositide 3-kinase/protein kinase B (PI3K/Akt) and mitogen-activated protein kinase (MAPK) signaling pathways as potentially associated in the osteogenic modulation of GQDs on hPDLSCs in both normal and inflammatory settings. Subsequent validation showed that GQDs upregulated the mRNA and total protein levels of p38, PI3K, and Akt. *In vivo*, local administration of GQDs attenuated alveolar bone resorption, suppressed osteoclast activity and formation, and reduced inflammatory infiltration with no observable systemic toxicity.

**Conclusion:**

GQDs possess the capacity to counteract inflammation-induced damage and promote osteogenic restoration both *in vitro* and *in vivo*, highlighting their potential dual functionality in periodontal regeneration.

## 1 Introduction

Chronic periodontitis, a widespread inflammatory oral disease associated with a pathogenic shift in the oral microbiome, progressively destroys periodontal supporting tissues, ultimately representing the foremost cause of tooth loss in adults (Slots, 2013; Trindade et al., 2023; Zhan et al., 2023). Despite being the standard of care, conventional non-surgical and surgical therapies frequently fall short of inducing substantial and reproductible alveolar bone regeneration (Zhibang et al., 2015). In light of this, periodontal tissue engineering has arisen as a promising regenerative paradigm. This strategy seeks to reconstruct the tissues through the synergistic application of osteoinductive factors, appropriate stem/progenitor cells, and advanced biomaterial scaffolds (Xu et al., 2019).

As a type of mesenchymal stem cell, human periodontal ligament stem cells (hPDLSCs) are a primary candidate for periodontal tissue engineering, possessing self-renewal ability, multipotency, low immunogenicity and the potential to differentiate into fibroblasts, cementoblasts, and osteoblasts under appropriate conditions (Eleuterio et al., 2013; Nuñez et al., 2019). Nevertheless, the inflammatory microenvironment in periodontitis severely undermines bone regeneration by dysregulating host immune responses and directly impairing the differentiation of hPDLSCs via disruption of endogenous signaling pathways. Herein, a major research focus is the identification of functional biomaterials that can counteract this inflammatory inhibition and rescue the osteogenic potential of hPDLSCs, which is essential for effective periodontal bone regeneration. Although the Porphyromonas gingivalis (*P. gingivalis*) lipopolysaccharide (LPS)-stimulated model is a simplified surrogate for the complex periodontal inflammatory milieu, it is widely accepted as a standard *in vitro* system for studying host-pathogen interactions, as *P. gingivalis* is a keystone pathogen and its LPS activates TLR4 signaling to release proinflammatory cytokines that mimic clinical conditions (Dauphinee and Karsan, 2006; Tang et al., 2016; Xu et al., 2020). This model provides a reproducible platform for initial investigation before more complex *in vivo* validation.

Multiple biomaterials, from bioceramics to alloys, have been applied to periodontal bone regeneration; yet their effectiveness is often limited by passive osteocompatibility rather than active osteoconduction (Shi et al., 2023). Graphene and its derivatives have recently garnered interest as an innovative regenerative platform due to their unique physicochemical properties (Tahriri et al., 2019). Notably, graphene quantum dots (GQDs) offer nanoscale dimensions, high surface functionality, good biosafety, favorable metabolic clearance, and optimal stability (Chong et al., 2014; Ema et al., 2017; Srimaneepong et al., 2022), along with reported abilities to promote stem cell osteogenic and adipogenic differentiation (Qiu et al., 2016; Wang et al., 2022). While our previous studies demonstrated that GQDs promote hPDLSCs migration and osteogenic differentiation under normal conditions (Gao et al., 2023; Liang et al., 2023), several knowledge gaps remain: (1) the global transcriptional changes included by GQDs in periodontitis have not been characterized; (2) the *in vivo* efficacy of GQDs in periodontitis models has not been validated; and (3) the signaling pathways associated with GQDs’ protective effects are largely unknown. The present study extends our prior work by providing transcriptomic profiling, *in vivo* efficacy validation in a periodontitis rat model, and initial association with certain signaling.

This study therefore seeks to address these unresolved issues by investigating the protective, anti-inflammatory, and osteogenic effects of GQDs *in vitro* and *in vivo* inflammatory models, and by identifying molecular pathways associated with the observed effects.

## 2 Materials and methods

### 2.1 *In vitro* effect of GQDs on hPDLSCs biological behaviors

#### 2.1.1 Cell isolation and identification

hPDLSCs were harvested from healthy premolars or third molars of donors (aged 18–25 years) at the Stomatological Hospital of Guangxi Medical University, following approved ethical protocols (No, 20200050). Informed consents were obtained from donors or legal guardians of any participants under the age of 18. Primary cells were isolated and expanded as in a preliminary study (Liang et al., 2023). Briefly, Samples were rinsed with Phosphate-buffered saline (PBS; Gibco, United States) supplemented with 1% penicillin/streptomycin (Gibco, United States). Periodontal tissues were minced into 1–3 mm^2^ explants and digested with 3 mg/ml type I collagenase (Sigma, United States) at 37 °C for 30 min. After centrifugation (1,200 rpm for 5 min), the supernatant was discarded, and the cell pellet was resuspended in alpha-modified Eagle’s minimum essential medium (α-MEM; Gibco, United States) containing 1% penicillin/streptomycin and 10% fetal bovine serum (FBS; Gibco, Grand Island, United States). Cells were cultured at 37 °C with 5%CO_2_. Medium was renewed every 3 days, and cells were passaged with trypsin-EDTA (0.25% trypsin; 0.02% EDTA; Wisent, Canada) at 70%–80% confluence. Cells at 3rd passage were applied to subsequent experiments.

The immunophenotype of hPDLSCs was analyzed using flow cytometry. Harvested cells were washed, centrifuged, and resuspended in PBS at a density of 1 × 10^6^ cells/ml. For immunostaining, 50 µl of the single-cell suspension was incubated with 5 µl each of CD34^−^fluorescein isothiocyanate (FITC), CD90^−^allophycocyanin (APC), CD146-peridinin-chlorophyll-protein complex-cyanine 5.5 (PerCP-Cy5.5), and STRO-1- phycoerythrin (PE) antibodies (Abcam, UK) for 20 min in the dark. Flow cytometric analysis was subsequently performed on a BD flow cytometer (BD Bio-science, United States).

To assess multilineage differentiation potential, hPDLSCs were planted in 6-well plates at a density of 2 × 10^4^ cells/well and maintained in complete medium. Upon reaching 60%–70% confluence, the medium was replaced with osteogenic induction medium (OIM; Cyagen, United States) to initiate osteogenic differentiation for 3 weeks. For adipogenic differentiation, confluent or post-confluent cells were switched to adipogenic induction medium (AIM; Cyagen, United States) for 3–4 weeks, with medium refreshed strictly following the manufacturer’s protocol. After induction, cells were fixed with 4% paraformaldehyde (Solarbio, China) for 30 min, following by staining with Alizarin Red S (ARS; Beyotime, China) or Oil Red O (Beyotime, China) to visualize mineralized nodules and lipid droplets, respectively.

#### 2.1.2 Characterization of GQDs

The GQDs (Nanjing XFNANO Materials Tech Co., Ltd., China; CAS: XF152-7440-44-0; Art.No.:102468) employed in the present study were derived from the identical commercial batch as that used in our earlier work (Liang et al., 2023), thereby ensuring material consistency; the detailed physicochemical properties of this batch have been thoroughly characterized and reported therein.

#### 2.1.3 Cell counting kit-8 (CCK-8) assay

The concentration of GQDs (5 μg/ml) was chosen based on our previous dose–response studies, which identified this concentration as optimal for promoting osteogenesis without compromising cell viability (Gao et al., 2023; Liang et al., 2023). An inflammatory microenvironment was established by supplementing the culture medium with 10 μg/ml P.g-LPS (InvivoGen, United States).

Cell viability was evaluated using the Cell Counting Kit-8 (CCK-8; Solarbio, China). hPDLSCs were seeded into 96-well plates at a density of 3 × 10^3^ cells/well and assigned to four groups: normal group (Norm; complete medium only), normal + GQDs group (Norm.+GQDs; complete medium containing 5 μg/ml GQDs), inflammation group (Infla.; complete medium containing 10 μg/ml P.g-LPS), and inflammation + GQDs (Infla.+GQDs; Complete Medium containing 10 μg/ml P.g-LPS and 5 μg/ml GQDs). All conditions were performed in triplicate biological replicates. After 24, 48 and 72 h of incubation, 10 µl of CCK-8 reagent (Solarbio, China) was added to each well, followed by incubation at 37 °C under 5% CO_2_ in the dark for an hour. Absorbance was measured at 450 nm using a microplate reader (TECAN Infinite M200Pro, canton of Zürich, Switzerland).

#### 2.1.4 Transwell assay

Cell migration was assessed using Transwell chambers and inserts (8.0 µm pore size; Corning, United States). hPDLSCs were seeded into the upper inserts at a density of 2 × 10^5^ cells/well in 100 µl serum-free medium. The lower chambers were filled with 700 µl of the corresponding medium (same grouping as in CCK-8). After 24 h of incubation at 37 °C under 5% CO_2_, non-migrated cells on the upper surface were carefully removed with a sterile cotton swab. Migrated cells were fixed with 4% paraformaldehyde and stained with 0.1% crystal violet solution (Solarbio, China) for 30 min. Images were acquired under an optical microscope (Olympus, Japan) and migrated cells were counted by two independent observers blinded to group allocation. The intraexaminer kappa value was >0.85.

#### 2.1.5 Alkaline phosphatase (ALP) staining and activity analysis

To assess the early osteogenic effects of GQDs, hPDLSCs were seeded in 12-well plates at a density of 2 × 10^4^ cells/well. Upon reaching 70%–80% confluence, cells were divided into 4 groups: normal group (Norm.; OIM only), normal + GQDs group (Norm.+GQDs; OIM containing 5 μg/ml GQDs), inflammation group (Infla.; OIM with 10 μg/ml P.g-LPS), and inflammation + GQDs group (Infla.+GQDs; OIM containing 5 μg/ml GQDs and 10 μg/ml P.g-LPS). After 7 days of induction, the culture medium was aspirated and cells were washed twice with PBS to remove residual GQDs, thereby minimizing any potential optical interference from GQDs’ absorption/fluorescence properties. For staining, cells were then fixed with 4% paraformaldehyde for 10 min and stained with BCIP/NBT Alkaline Phosphatase Color Development Kit (Beyotime, China). For activity assay, parallel cultures were also washed twice with PBS, then lysed with 200 µl RIPA buffer (Beyotime, China). Lysates were centrifuged at 12,000 × *g* at 4 °C for 5 min, and the supernatant was analyzed using an Alkaline Phosphatase Assay Kit (Beyotime, China) according to the manufacturer’s instructions.

#### 2.1.6 Alizarin red s (ARS) staining and semi-quantitative analysis

Matrix mineralization was assessed after 21 days of induction (same grouping as ALP analysis). The culture medium was aspirated and cells were washed twice with PBS to remove residual GQDs, thereby minimizing any potential optical interference from GQDs’ absorption/fluorescence properties. For staining, cells were then fixed with 4% paraformaldehyde and stained with ARS solution for 5 min at room temperature. After staining, cells were gently rinsed with distilled water. For semi-quantitation, stained nodules were dissolved in 10% Cetylpyridinium chloride (Aladdin, Shanghai, China) solution for 30 min with gentle agitation, and absorbance was measured at 562 nm by a microplate reader.

#### 2.1.7 Enzyme linked immunosorbent assay (ELISA)

The anti-inflammatory effect of GQDs was detected by ELISA. hPDLSCs (2 × 10^5^ cells/well) were seeded in 6-well plates and subjected to the same grouping as in CCK-8 assay. After 7 days of culture, supernatants were collected and centrifugated at 1,000 rpm for 5 min at room temperature. Levels of interleukin-1beta (IL-1β), interleukin-6 (IL-6), and tumor necrosis factor-alpha (TNF-α) were quantified using commercial ELISA kits (MEIMIAN, China) according to the manufacturer’s protocols.

#### 2.1.8 mRNA-sequencing (m-RNA seq)

To investigate potential osteogenic mechanisms, mRNA-Seq was performed on hPDLSCs under the same osteogenic induction as ALP analysis. Total RNA was isolated using an RNA extraction kit (Seven Biotech, China). RNA quality assessment, library construction, and Illumina sequencing (Novaseq 6000, PE150) were conducted by Genedenovo Biotechnology Co., Ltd. (Guangzhou, China). Three biological replicates per condition were used. Differentially expressed genes (DEGs) were identified with thresholds of |log2FC|≥1 and adjusted p-value ≤ 0.05 (Benjamini-Hochberg FDR). Functional enrichment analysis was performed using Gene Ontology (GO) terms and Kyoto Encyclopedia of Genes and Genomes (KEGG) pathways, with hypergeometric tests and FDR correction.

#### 2.1.9 Real-time quantitative polymerase chain reaction (RT-qPCR)

To correlate with mRNA-Seq results, the mRNA expression levels of mitogen-activated protein kinase p38 (p38), phosphoinositide 3-kinase (PI3K), and protein kinase B (Akt) were quantified by RT-qPCR. Total RNA was extracted using TRIzol reagent (Thermo Fisher, United States). First-strand cDNA was synthesized with Hiscript III qRT SuperMix (Vazyme, China). RT-qPCR was performed in triplicate using ChamQ SYBR qPCR Master Mix (Vazyme, China) on a QuantStudio5 Real-Time PCR System (Thermo Fisher, United States). Relative gene expression was calculated via the 2^−ΔΔCt^ method. GAPDH was used as the endogenous reference gene, as its Ct values remained stable across all experimental groups in our preliminary assessment (coefficient of variation <5%). Primer efficiency was confirmed to be 90%–110% for all targets, and melt curve analysis showed single peaks. Primer sequences are provided in Table 1.

**TABLE 1:** Primer sequences of each gene.

Name	Forward (5′-3′)	Reverse (5′-3′)
GAPDH	TCT​CCT​CTG​ACT​TCA​ACA​GCG​ACA	CCC​TGT​TGC​TGT​AGC​CAA​ATT​CGT
p38	CTG​GCT​CGG​CAC​ACA​GAT​GAT​G	GCC​TCA​TGG​CTT​GGC​ATC​CTG
PI3K	AGC​CTT​CCT​CTG​TGT​CCC​TGT​G	CTT​GGT​GGA​GCG​AGC​CTT​CTT​G
Akt	GCT​GGG​CAA​GGG​CAC​TTT​CG	GAG​GCG​GTC​GTG​GGT​CTG​G

#### 2.1.10 Western blot (WB) assay

Western blot was performed to assess total protein levels of PI3K, Akt, and p38. Total protein was extracted by RIPA lysis buffer (Beyotime, China) and quantified with a BCA Protein Assay Kit (Beyotime, China). Protein samples were separated by 12.5% sodium dodecyl sulfate polyacrylamide gel electrophoresis (SDS-PAGE) gel (Epizyme, China) and transferred onto polyvinylidene fluoride (PVDF) membranes (Servicebio, China). After blocking with 5% (w/v) skimmed milk for 1 h at room temperature, membranes were incubated overnight at 4 °C with primary antibodies against p38, Akt, and PI3K (ZEN-Bio, China, 1:500 dilution). β-tubulin was used as the loading control, as its expression levels were consistent across all samples, with less than 10% variation in densitometric analysis. Following washing with Tris-buffed saline with Tween 20 (TBST, Solarbio, China), membranes were incubated with horseradish peroxidase (HRP)-conjugated secondary antibodies (ZEN-Bio, China, 1:10,000 dilution) for 1 h at room temperature. Protein bands were visualized using enhanced chemiluminescence (ECL) reagents (SEVEN, China) and imaged with a ChemiDoc Touch System (BIO-RAD, United States).

### 2.2 *In vivo* alveolar bone repair efficacy and biosafety of gqds analysis

#### 2.2.1 Establishment of experimental rat periodontitis model

All animal procedures were approved by the Ethical Committee of Guangxi Medical University (No: 202306009) and were conducted in accordance with the ARRIVE 2.0 guidelines (Percie du Sert et al., 2020). Twenty 7-week-old male Sprague-Dawley (SD) rats (200–220 g) were obtained from the Experimental Animal Center of Guangxi Medical University and housed under specific-pathogen-free (SPF) conditions with a 12 h light/dark cycle and free access to food and water. After 1 week of acclimatization, periodontitis was induced in the maxillary first molars by placing a 3–0 silk suture ligature into gingival sulcus. Surgery was performed under general anesthesia induced by intraperitoneal injection of 1% sodium pentobarbital (40 mg/kg; ZENOAQ, Japan). To minimize intraoperative pain and bleeding, local infiltration of 2% lidocaine containing 1:80,000 epinephrine (4 mg/kg; ZENOAQ, Japan) was administered. Ligatures were checked daily and promptly replaced if dislodged. Humane endpoints were defined as ≥20% body weight loss, inability to access food and water, or severe distress, at which point animals would be immediately euthanized; no animal reached these endpoints during this study.

After 4 weeks, ligature induced periodontitis was confirmed by clinical examination (bleeding probing, pocket formation). Sample size (n = 5 per group) was determined based on previous studies with similar rat periodontitis models and graphene-based nanomaterials, which have shown that five animals per group are sufficient to detect statistically significant differences in bone loss (An et al., 2024). Although no formal power analysis was conducted, the magnitude of the observed effects and the variance within groups confirmed that this sample size was adequate for the study. Rats were then randomly divided into four groups using a computer-generated random number sequence: blank group (healthy, no ligation), periodontitis group (ligation only), saline group (ligation, weekly local injection of 50 μl saline solution), experimental group (ligation, weekly local injection of GQDs solution at 50 μl of 5 μg/ml in sterile saline). Randomization was performed using a computer-generated sequence by an investigator not involved in the subsequent experiments. Allocation was concealed using sealed envelopes. The *in vivo* dose was extrapolated from the *in vitro* effective concentration, with consideration of the maximal injectable volume (50 μl) in the rat periodontal pocket, corresponding to 0.25 µg GQDs per site; however, we recognize that pharmacokinetic and biodistribution studies are needed to establish the optimal regimen in future. All local injection were administered into the periodontal pocket using a micro-syringe. At the end of the 4-week treatment period, rats were euthanized by intraperitoneal injection of sodium pentobarbital at a dose of 150 mg/kg–more than three times the anesthetic dose–to ensure rapid and painless death. The absence of heartbeat and respiration was confirmed before tissue collection. All euthanasia procedures followed the AVMA Guidelines for the Euthanasia of Animals (Underwood, 2020). Maxillae were harvested and fixed in 10% neutral buffered formalin.

#### 2.2.2 Micro-computed tomography (Micro-CT) and three-dimensional reconstruction

The harvested maxillae were scanned using a high-resolution micro-CT system (Hitachi, Japan) with the following paraments: voltage 85 kV, current 114 μA, and isotropic voxel size 5 μm. Three-dimensional (3D) reconstructions were generated using Mimics Research software (v21.0.0, Materialise, Belgium). Alveolar bone resorption was quantified by measuring the linear distance from the cemental-enamel junction (CEJ) to the alveolar bone crest (ABC) at six predetermined sites per tooth, including mesiobuccal, distobuccal, mesiopalatal, distopalatal, mid-buccal, and mid-palatal. Measurement were performed by a single trained observer blinded to group allocation.

#### 2.2.3 Histological analysis

Maxillae were decalcified in 10% ethylenediaminetetraacetic acid (EDTA) at 4 °C for 4 weeks, followed by dehydration in a graded ethanol series, paraffin embedding, and sectioning at 5 µm thickness. Consecutive sections were stained using commercial kits according to manufacturers’ instructions: Hematoxylin and Eosin (H&E; Solarbio, China) for general morphology, Masson’s Trichrome (Solarbio, China) for collagen visualization, and Tartrate Resistant Acid Phosphatase (TRAP; Solarbio, China) for osteoclast detection. Multinucleated cells (≥3 nuclei) exhibiting TRAP positivity were identified as osteoclasts.

#### 2.2.4 Biosafety detection

To evaluate the system biosafety of GQDs, major organs including heart, liver, spleen, lungs, and kidneys were harvested for H&E staining.

### 2.3 Statistical analysis

SPSS 25.0 (IBM, United States) software and GraphPad Prism 9 (GraphPad Software, United States) were applied for statistical analysis. Normality was assessed using the Shapiro–Wilk test. Homogeneity of variances was tested using Levene’s test. For comparisons among four groups, one-way ANOVA followed by Tukey’s post-hoc test was applied when variances were homogeneous and data were normally distributed; otherwise, the Kruskal-Wallis test with Dunn’s post-hoc test was used. All *p*-values are two tailed, *p*-value < 0.05 is indicated that statistically difference. All experiments were repeated in three independent biological replicates with technical triplicates. Data are presented as mean ± SD.

## 3 Results

### 3.1 Isolation, culture and characterization of hPDLSCs

The isolated hPDLSCs exhibited characteristic spindle-shaped morphology, expressed mesenchymal stem cell markers (CD90^+^, CD146^+^, STRO-1^+^) and were negative for the hematopoietic marker CD34^−^, and demonstrated multilineage differentiation potential toward osteogenic and adipogenic lineages (Supplementary Figure S1). These findings confirm that the isolated cells meet the minimal criteria for mesenchymal stem cells.

### 3.2 Cell viability and migration analysis

**FIGURE 1:**
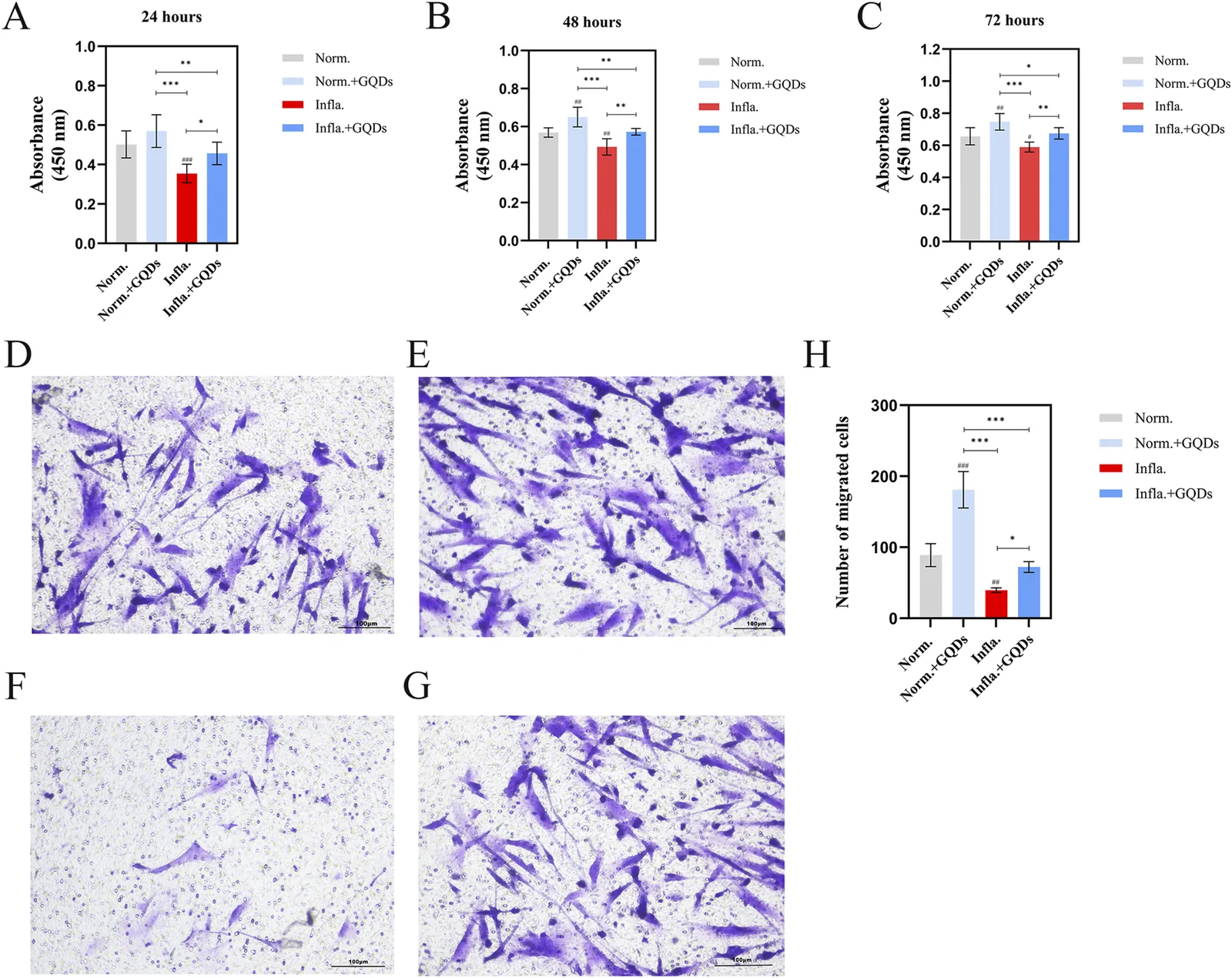
CCK-8 and Transwell assays. **(A–C)** Cytotoxicity of GQDs on hPDLSCs, **(A)** 24 h, **(B)** 48 h, **(C)** 72 h. **(D–H)** Migration of hPDLSCs, **(D)** normal group; **(E)** normal + GQDs group; **(F)** inflammation group; **(G)** inflammation + GQDs group; **(H)** Statistical analysis of the migrated cells number. Data are represented as mean ± SD of three independent experiments. *p < 0.05, **p < 0.01, ***p < 0.001. # indicates a statistically significant difference compared to the normal group, ^#^ *p* < 0.05, ^##^ *p* < 0.01, ^###^ *p* < 0.001.

CCK-8 assay showed that GQDs significantly enhanced hPDLSCs proliferation under normal conditions at 48 h and 72 h (*p* < 0.01), whereas no significant difference was observed at 24 h (*p* > 0.05). Conversely, P.g-LPS induced inflammation markedly reduced cell viability (*p* < 0.05). Importantly, GQDs restored proliferation in the inflammatory microenvironment, reversing this suppression (*p* < 0.05) (Figures 1A–C). As shown in Figures 1D–H, Transwell assay indicated that GQDs promoted hPDLSCs migration in normal microenvironment (*p* < 0.001; Figure 1E), whereas P.g-LPS substantially inhibited migration (*p* < 0.05; Figure 1F). Notably, GQDs partially rescued migration capacity under inflammatory circumstance (*p* < 0.05; Figure 1G).

### 3.3 Osteogenic differentiation assessments

**FIGURE 2:**
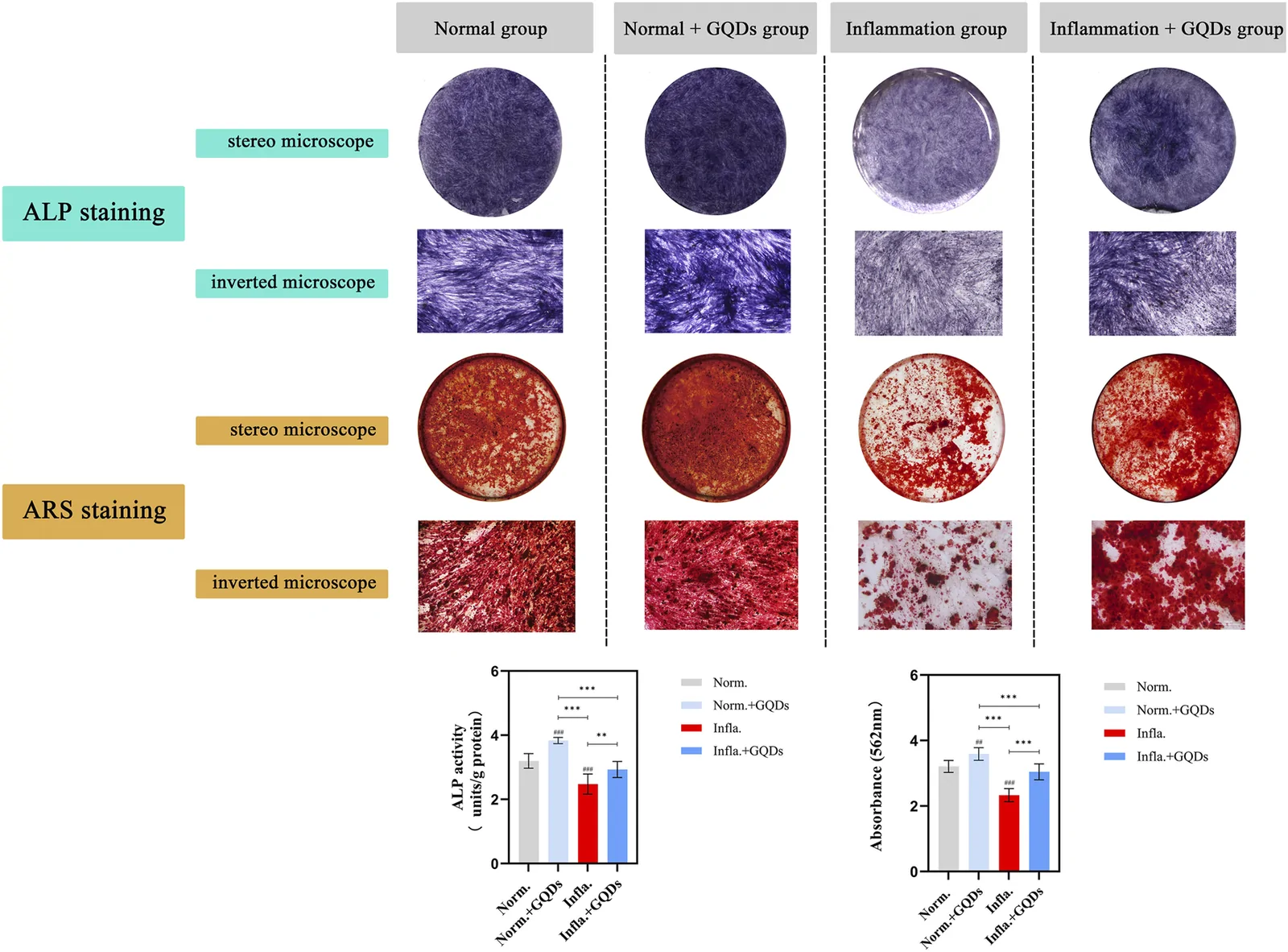
Osteogenic differentiation assessments. Data are represented as mean ± SD of three independent experiments. *p < 0.05, **p < 0.01, ***p < 0.001. # indicates a statistically significant difference compared with the normal group, ^#^ *p* < 0.05, ^##^ *p* < 0.01, ^###^ *p* < 0.001.

As illustrated in Figure 2, GQDs significantly enhanced early osteogenic differentiation under normal conditions, evidenced by increased ALP activity and more intense blue staining (*p* < 0.001).Similarly, ARS staining and semi-quantitation revealed a marked increase in mineralized nodule formation by GQDs in normal environment (*p* < 0.01). Under inflammatory conditions, both ALP activity and mineralized nodule formation were significantly suppressed (*p* < 0.001). However, GQDs effectively counteracted this inhibition, restoring both ALP activity (*p* < 0.01) and mineralized nodule formation in the inflammatory conditions (*p* < 0.001). These results demonstrate that GQDs promote the osteogenic differentiation of hPDLSCs under normal conditions and alleviate inflammation induced impairment.

### 3.4 Enzyme-linked immunosorbent assay (elisa) analysis

**FIGURE 3:**
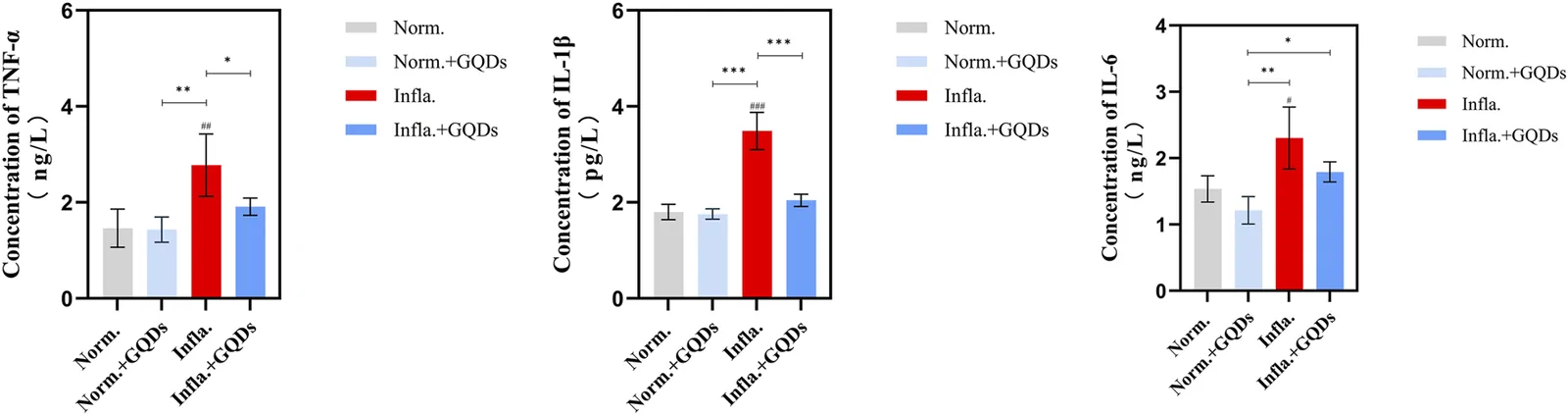
Enzyme-linked immunosorbent assay. Data are represented as mean ± SD of three independent experiments. *p < 0.05, **p < 0.01, ***p < 0.001. # indicates a statistically significant difference compared with the normal group, ^#^ *p* < 0.05, ^##^ *p* < 0.01, ^###^ *p* < 0.001.

Under normal circumstances, GQDs did not significantly alter the secretion of TNF-α, IL-1β and IL-6 compared with the normal group (*p* > 0.05). In contrast, P.g-LPS stimulation markedly elevated the levels of TNF-α (*p* < 0.01), IL-1β (*p* < 0.001) and IL-6 (*p* < 0.05) compared with the normal group. whereases GQDs notably attenuated this inflammatory response, reducing TNF-α (*p* < 0.05), IL-1β(*p*<0.001) in inflammatory circumstance, while there was no significant difference in IL-6 levels between inflammation group and inflammation + GQDs group (Figure 3).

### 3.5 Transcriptomic analysis of osteogenic mechanisms

**FIGURE 4:**
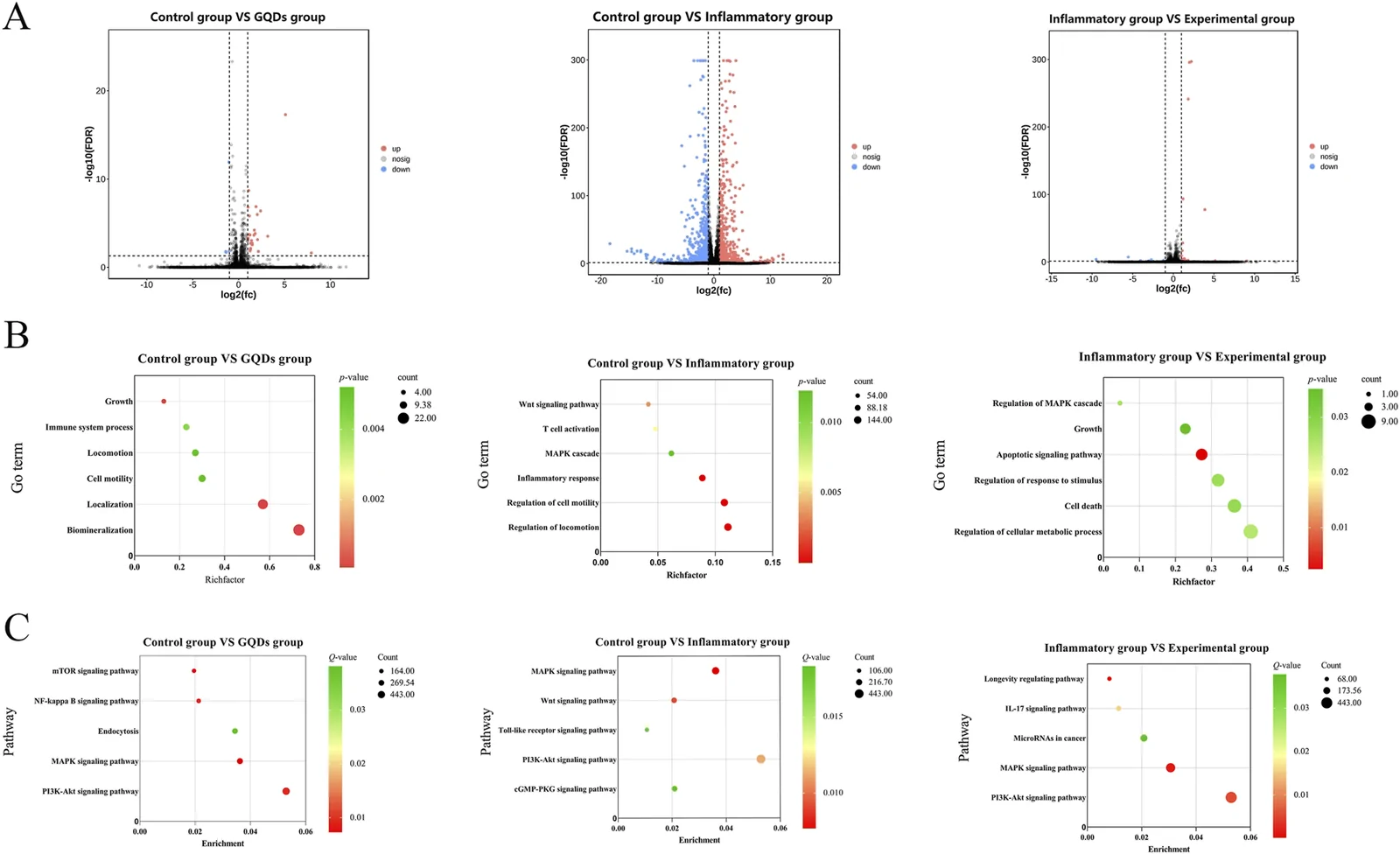
Transcriptome sequencing analysis. **(A)** Differential expression analysis. **(B)** Gene ontology enrichment analysis. **(C)** Kyoto encyclopedia of genes and genomes analysis.

As shown in Figure 4, the relatively modest transcriptional changes induced by GQDs (4 up- and 26 downregulated genes under normal conditions; 8 up- and 19 downregulated under inflammatory conditions) compared with the extensive alterations triggered by P.g-LPS stimulation (708 up- and 700 downregulated genes) suggest that GQDs exert a targeted modulatory effect rather than a global transcriptional reprogramming. This is consistent with the notion that GQDs may fine-tune specific signaling pathways to restore osteogenic homeostasis without broadly disrupting cellular functions. GO enrichment indicated that the effect of GQDs in normal circumstance was associated with processes including cell growth, motility, biomineralization, and immune regulation system processes. The modulation of GQDs under inflammatory conditions was related to cell proliferation, apoptosis, MAPK regulation, and metabolic process. KEGG pathway analysis further demonstrated distinct signaling profiles that the effect of GQDs in normal circumstance was enriched in mTOR, NF-κB, endocytosis, MAPK, and PI3K/Akt pathways. The modulation of GQDs under inflammatory conditions was involved in longevity-regulating, IL-17, micro-RNA in cancer, MAPK, and PI3K/Akt pathways. Collectively, these transcriptomic data suggest a potential association for MAPK and PI3K/Akt signaling in the response to GQDs.

**FIGURE 5:**
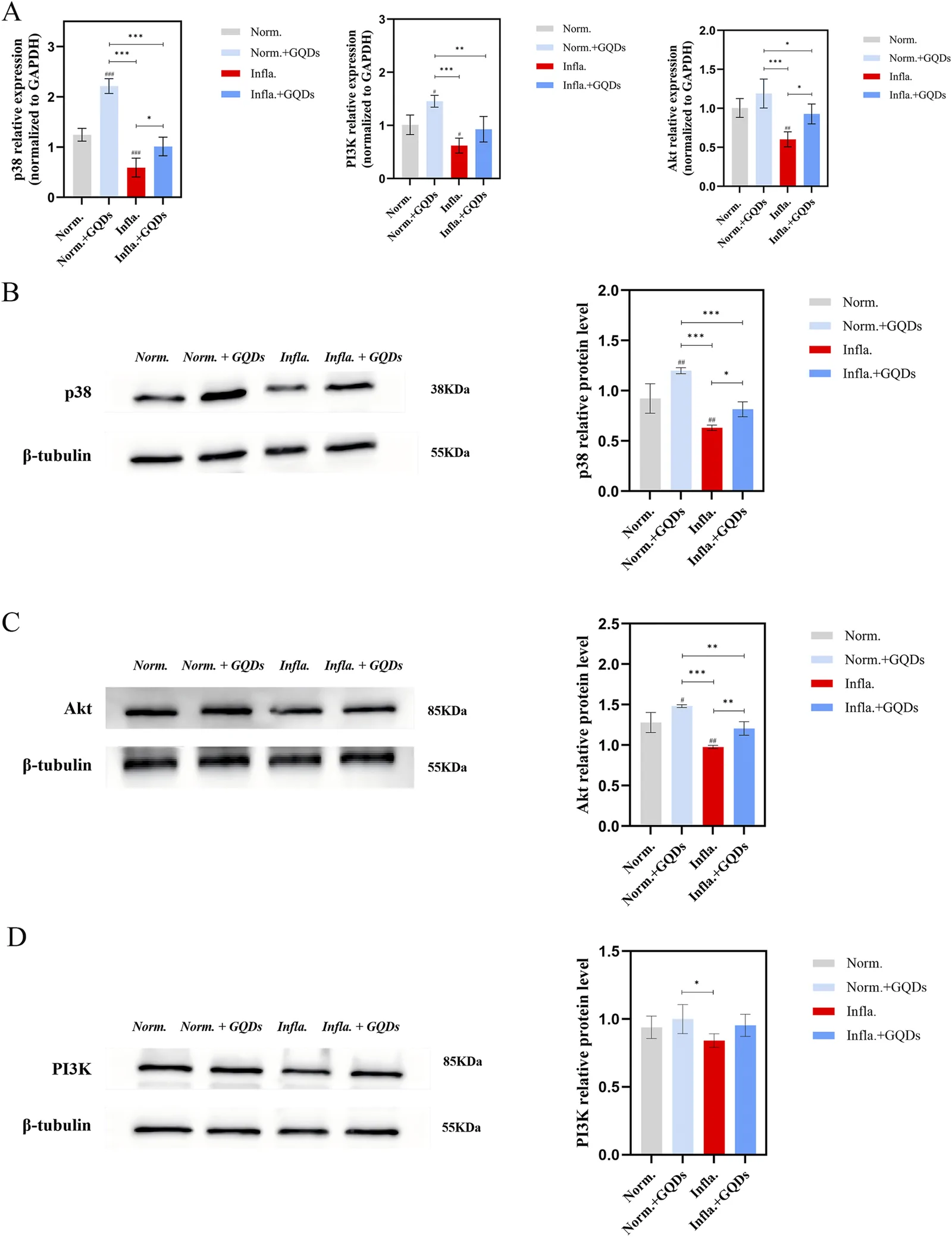
Quantitative Real-time PCR and Western blot analysis **(A)** PCR analysis; **(B–D)** western bolt analysis. Data are represented as mean ± SD of three independent experiments. *p < 0.05, **p < 0.01, ***p < 0.001. #indicates a statistically significant difference compared with the normal group, ^#^ *p* < 0.05, ^##^ *p* < 0.01, ^###^ *p* < 0.001.

As shown in Figure 5A, GQDs significantly upregulated the mRNA expression of p38 (*p* < 0.001) and PI3K (*p* < 0.05) under normal microenvironment, while no statistically significant difference in Akt. In contrast, inflammation markedly downregulated p38 (*p* < 0.001), PI3K (*p* < 0.05), and Akt (*p* < 0.01). Importantly, GQDs restored genes expression of p38 and Akt under inflammation stress relative to the inflammation group (*p* < 0.05). At the protein level, WB further confirmed that GQDs increased total protein level of p38 (*p* < 0.05), and Akt (*p* < 0.01) under normal circumstance and reversed their inflammation-induced suppression, while there was no statistically significant difference in total PI3K protein level (Figures 5B–D). As phosphorylation status was not assessed, these results indicate increased total protein levels rather than pathway activation, identifying the PI3K/Akt and MAPK signaling pathways as potentially associated in the biological modulation of GQDs on hPDLSCs in both normal and inflammatory settings.

### 3.6 *In vivo* bone repair effect and biosafety analysis

Throughout the 4-week ligation period, all animals maintained normal general health, with stable food/water intake and physiological weight gain. Periodontitis was successfully induced, as confirmed by ligature presence, localized gingival inflammation and swelling, and visible plaque accumulation around the maxillary first molars. Bleeding on probing and periodontal pocket formation further validated the periodontitis rat model.

**FIGURE 6:**
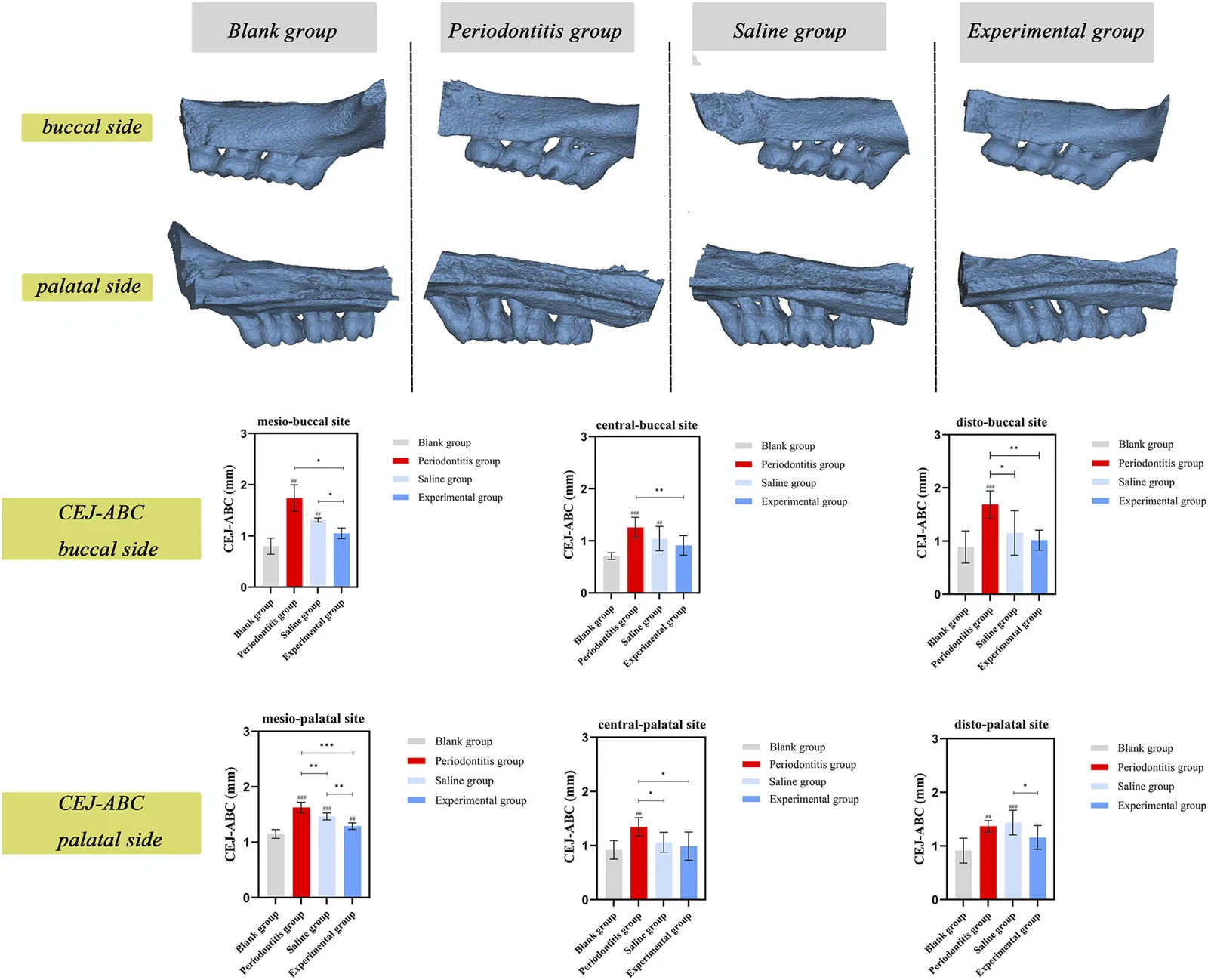
Micro-CT and CEJ-ABC analysis. Data are represented as mean ± SD of three independent experiments. *p < 0.05, **p < 0.01, ^a^ statistical difference compared to blank group.

As shown in Figure 6, Micro-CT reconstructions revealed distinct alveolar bone morphology among groups. The blank group exhibited intact alveolar bone with no notable resorption. The periodontitis and saline groups showed pronounced bone loss on both buccal and palatal aspects, particularly in the distal regions where arc-like resorption extended over half the root length. Linear CEJ-ABC distances at multiple sites were significantly greater in periodontitis and saline groups than in the blank group (*p* < 0.01). Notably, the experimental group displayed markedly attenuated bone loss, with resorption limited to less than one-third of the root length. Although the CEJ-ABC distance at the mesiopalatal site remained greater than in the blank group (*p* < 0.01), it was significantly reduced at the mesiobuccal, mesiopalatal, and central-buccal/palatal sites relative to the periodontitis group (*p* < 0.01), and at mesiobuccal, mesiopalatal, and distopalatal sites compared with the saline group (*p* < 0.05).

**FIGURE 7:**
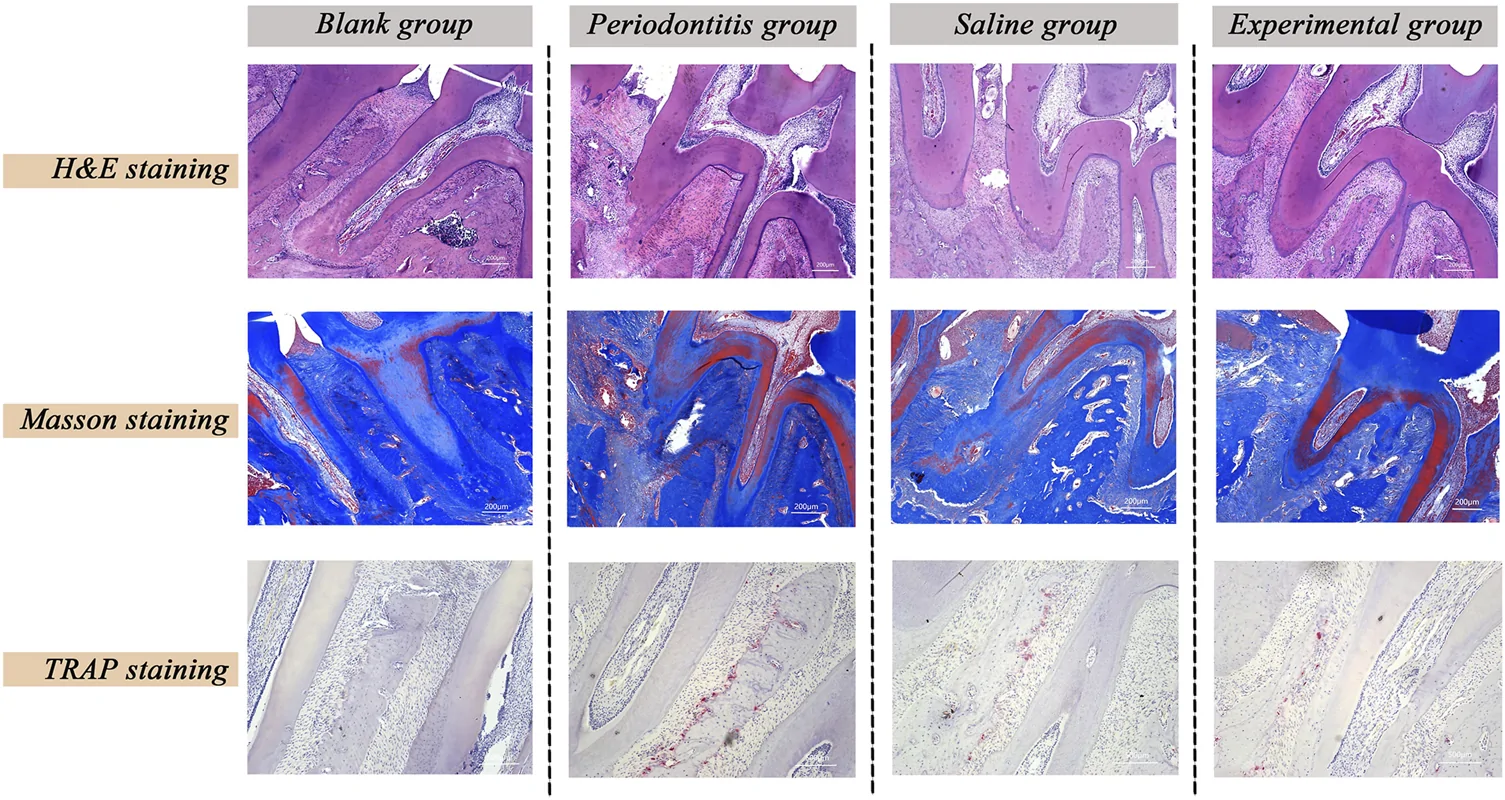
Histological staining.

H&E staining showed well-preserved periodontal architecture in the blank group, with regular epithelial thickness, densely arranged collagen fibers, and an intact alveolar crest. The periodontitis group displayed severe destruction, including significant alveolar bone resorption, irregular bone surface, and extensive inflammatory infiltration. The saline group showed similar but milder changes. Notably, the experimental group exhibited markedly attenuated inflammatory infiltration, only mild epithelium disruption, and considerable improved tissue integrity (Figure 7).

Masson’s trichrome staining demonstrated well-oriented, normally attached periodontal ligament fibers in the blank group. In contrast, periodontitis and saline groups showed disorganized, sparse, and degenerated collagen fibers. GQDs treatment significantly improved collagen organization, with reappearance of dense, well-aligned blue-stained fibers and red-stained bone-like tissues along the root surface (Figure 7).

TRAP staining showed no TRAP-positive osteoclasts or resorption lacunae in the blank group. Periodontitis and saline groups presented abundant TRAP-positive cells (stained red) and multiple resorption lacunae along the alveolar bone surface. The experimental group exhibited a marked reduction in TRAP-positive cells and resorption lacunae (Figure 7).

**FIGURE 8:**
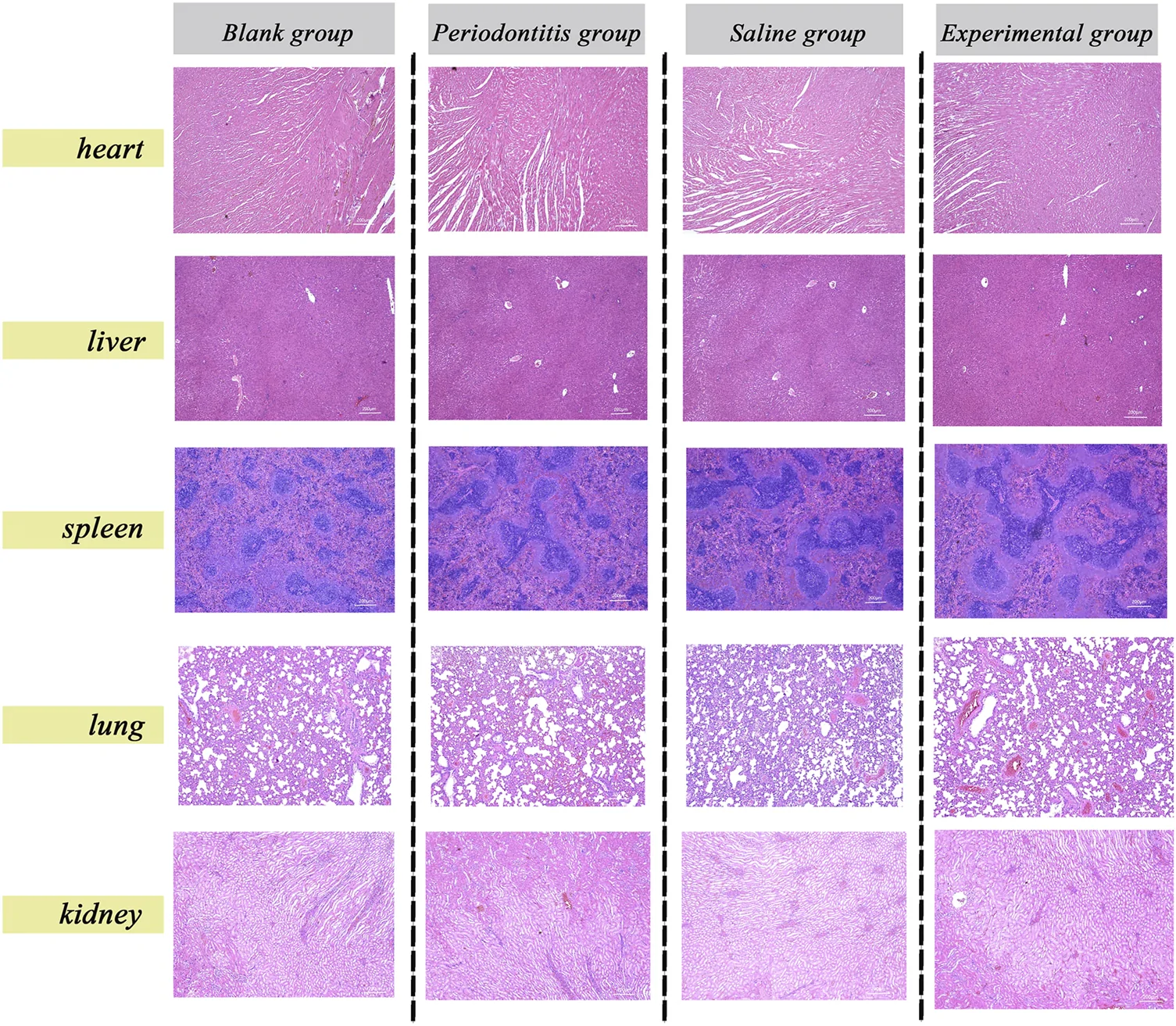
Biosafety assessment (H&E staining) of GQDs *in vivo*.

HE staining of heart, liver, spleen, lungs and kidneys showed no structural abnormalities or signs of inflammation following local GQDs administration (Figure 8). All organs retained normal histological architecture.

## 4 Discussion

Developing effective biomaterials that promote alveolar bone regeneration within an inflammatory microenvironment remains a central challenge in periodontitis. While our previous studies demonstrated that GQDs promote hPDLSCs migration and osteogenic differentiation under normal conditions and partially counteract inflammatory suppression, the present work extends these findings in three important aspects. First, we indicate that GQDs attenuate *in vitro* inflammation-mediated suppression of hPDLSCs viability, migration and osteogenesis and provide the transcriptomic characterization of GQDs-treated hPDLSCs, revealing the global gene expression landscape associated with GQDs exposure. Second, we validate the *in vivo* efficacy of locally administered GQDs in attenuating alveolar bone loss in a ligature-induced rat periodontitis model. Third, we establish an initial correlation between GQDs treatment and key osteogenic signaling components (p38, PI3K, Akt) at both the mRNA and total protein levels. Collectively, these integrated *in vitro* and *in vivo* investigations substantially advance our understanding of GQDs from a purely *in vitro* osteo-promotive agent toward a candidate therapeutic nanomaterial with demonstrated efficacy in a clinically relevant animal model.

hPDLSCs are considered a promising cell source for periodontal regeneration (Atsushi et al., 2019). Nevertheless, the inflammatory microenvironment in periodontitis is known to severely impair stem cell functions (Jakovljevic et al., 2018; Li et al., 2020; Maruyama et al., 2020; Kim, 2021; SenGupta et al., 2021; Gu et al., 2022). Our study provides direct experimental evidence that the inflammatory milieu significantly increased IL-1β, IL-6, and TNF-α levels and compromises hPDLSCs viability, migration, and osteogenic differentiation, underscoring the need for protective strategies.

Graphene-based nanomaterials have attracted broad interest in biomedical fields due to their favorable biocompatibility and ability to support stem cell proliferation (Kim et al., 2015; Zhao et al., 2019). As a zero-dimensional derivative, GQDs retain advantageous physicochemical properties while offering enhanced biocompatibility and high surface to volume ratio (Ema et al., 2017). Prior studies, including work on bone marrow-derived mesenchymal stem cells, have shown that GQDs can stimulate osteogenic markers such as ALP under normal circumstance (Qiu et al., 2016), consistent with our observations in hPDLSCs. This osteo-promotive effect may be attributed to the large specific surface area of GQDs, which can adsorb bioactive molecules and create a favorable microenvironment (Wei et al., 2017). Importantly, the present study extends these findings into an inflammatory setting. We demonstrate that GQDs not only reduced proinflammatory cytokine levels but also counteracted the detrimental impact of inflammation on hPDLSCs viability, migration, and osteogenesis. Given that periodontal tissues in patients exhibit elevated proinflammatory cytokines that suppress regeneration and drive bone loss (Deo and Bhongade, 2010), the anti-inflammatory capacity of GQDs likely represents a critical mechanism. It is noteworthy that while GQDs significantly reduced the elevated levels of TNF-α and IL-1β induced by P.g-LPS, the reduction in IL-6 levels did not reach statistical significance. This differential regulation may be attributed to distinct transcriptional regulatory mechanisms governing these cytokines. Although TNF-α, IL-1β, and IL-6 are all downstream effectors of NF-κB and MAPK signaling, their gene expression is also modulated by additional pathway-specific transcription factors (Liu et al., 2017). For instance, IL-6 expression is critically dependent on the JAK-STAT3 pathway and C/EBPβ, whereas TNF-α and IL-1β are more directly regulated by NF-κB (Tanaka et al., 2014; Hirano, 2021). The concentration of GQDs (5 μg/ml) or the treatment duration may have been insufficient to fully reverse IL-6 transcription, which requires a more sustained suppression of STAT3 activation. Alternatively, the IL-6 promoter contains multiple regulatory elements that may render it less sensitive to the anti-inflammatory effects of GQDs compared with TNF-α and IL-1β (Kang et al., 2019). These findings suggest that while GQDs exert broad anti-inflammatory activity, their effects on individual cytokines are not uniform, and further studies are warranted to dissect the specific molecular pathways underlying the differential regulation of these proinflammatory mediators.

The osteogenic differentiation of hPDLSCs by biomaterials involves a complex interplay of signaling pathways (Qiuyue et al., 2023). While the p38/MAPK pathway is well-established in osteogenesis (Wang et al., 2018; Ma et al., 2022), and the PI3K/Akt pathway plays momentous roles in cell survival, metabolism, and differentiation (Li et al., 2017; Ge et al., 2019), the specific mechanisms through which GQDs promote osteogenesis in hPDLSCs have remained unclear. In this study, transcriptomic profiling suggested that GQDs may be associated with these pathways. Subsequent experiments confirmed that GQDs upregulate mRNA and total protein levels of p38, PI3K, and Akt under normal and inflammatory conditions. It is important to note that our mechanistic findings are correlational rather than causal. While transcriptomic and total protein data point to an association between GQDs treatment and components of the MAPK and PI3K/Akt pathways, we did not assess phosphorylation status, nor did we perform pathway inhibition or rescue experiments. Therefore, we refrain from concluding that GQDs “activate” these pathways; instead, our data provide a foundation for future studies employing phospho-specific antibodies and pharmacological inhibitors. The present results should be interpreted as identifying candidate pathways associated with the osteogenic effects of GQDs, rather than establishing definitive mechanistic causality. Our findings indicate that GQDs are associated with increased expression of these signaling components, providing a foundational correlation that warrants further mechanistic dissection. Importantly, these results offer the transcriptomic and protein-level evidence linking GQDs to key osteogenic pathways in an inflammatory context, thereby guiding future studies toward targeted validation.

Inflammatory processes actively disrupt alveolar bone homeostasis, driving the characteristic bone loss in periodontitis. Our *in vivo* results indicate that local application of GQDs attenuated alveolar bone resorption in a rat periodontitis model, with no observed systemic toxicity. While no histopathological abnormalities were observed, histological evaluation alone is not sufficient to establish comprehensive biosafety. Longer-term studies with serum biochemistry and biodistribution are required. The histological reduction in inflammatory cell infiltration corroborates the anti-inflammatory activity of GQDs *in vitro*. Periodontitis-associated bone destruction stems from an imbalance between osteoblastic formation and osteoclastic resorption. As osteoclasts degrade the periodontal connective and junctional tissues, the resulting anatomical disruption hinders osteoblasts-mediated regeneration (Usui et al., 2021). Our study found that GQDs prominently suppressed osteoclasts formation and activity, suggesting that pro-regenerative effect of GQDs may involve the restoration of physiological osteoclast-osteoblast coupling. It should be noted that the histological evaluations in this study were performed qualitatively; quantitative histomorphometric analysis of inflammatory infiltration, collagen organization, and osteoclast activity were not conducted. Such quantitative assessments represent an important direction for future studies to provide more objective and robust evidence for the tissue-level effects of GQDs.

In summary, this study demonstrates the potential dual functionality of GQDs in promoting osteogenesis and exerting anti-inflammatory effects. GQDs reversed the inflammatory impairment of hPDLSCs viability, migration, and osteogenic differentiation *in vitro*, and this effect may correlate MAPK and PI3K/Akt pathways. *In vivo*, local GQDs administration attenuated inflammatory infiltration and alveolar bone loss by modulating osteoclasts activity, with favorable biosafety profile under the current experimental conditions. However, several limitations of this study should be acknowledged. First, the GQDs concentration and dose were empirically selected without systematic pharmacokinetic optimization. Second, the *in vitro* model uses a single bacterial component (P.g-LPS) and the *in vivo* model is a ligature-induced acute periodontitis, which does not fully replicate human chronic periodontitis. Third, our mechanistic analysis is limited to total protein levels without phosphorylation assessment or pathway inhibition, thus our conclusions are correlational. Moreover, while we observed consistent expression of GAPDH and β-tubulin across experimental conditions, the use of a single reference gene for qPCR and a single loading control for Western blot represents a technical limitation. Future studies employing multiple validated reference genes and loading controls would further strengthen the quantitative reliability of gene and protein expression analyses. Fourth, biosafety evaluation was confined to histology; comprehensive toxicological assessment requires additional biochemical and long-term studies. Future studies incorporating systematic dose optimization, phosphorylation-specific analyses with pathway inhibitor/rescue experiments, longer-term observation periods, and comprehensive biosafety evaluations including biochemical and biodistribution assessments will be essential to address these limitations and to further validate the translational potential of GQDs for periodontal regeneration.

## 5 Conclusion

GQDs effectively mitigate inflammation-induced impairment of hPDLSCs functions *in vitro* and attenuate alveolar bone resorption in a rat periodontitis model *in vivo*. The pro-osteogenic effects of GQDs are associated with increase expression of total p38, PI3K, and Akt, suggesting potential correlation with MAPK and PI3K/Akt signaling components. However, as phosphorylation status and functional pathway activation were not assessed, these findings should be interpreted as associations rather than mechanistic demonstrations. While the results highlight GQDs as a promising candidate for periodontal applications, longer-term studies, dose optimization, comprehensive safety evaluation, and mechanistic validation are required before clinical translation.

## Data Availability

The data that support the findings of this study are available upon request. The data includes: load data, raw image data and statistical analysis data, and will be made available by the corresponding author RQ upon request.
