# Supplementary material for: Graphene quantum dots remodel osteogenesis of human periodontal ligament stem cells under inflammatory microenvironment: an in vitro and in vivo study
Source: Front Bioeng Biotechnol. 2026 Aug 26;14:1879913. doi: 10.3389/fbioe.2026.1879913 (PMC13553934; doi:10.3389/fbioe.2026.1879913)
Supplement: Supplementary file 1 [file Supplementaryfile1.docx]

**Supplementary Materials S1**

*Figure S1 Isolation, culture, and characterization of hPDLSCs. (A) microphotography of primary periodontal ligament cells (Scale bar: 500μm); (B) colony culture of hPDLSCs stained with Crystal Violet (Scale bar: 200μm); (C) microphotography of representative data for Alizarin red-S staining of hPDLSCs differentiates into osteoblasts (Scale bar: 200μm); (D) microphotography of representative data for Oil Red O staining of hPDLSCs differentiates into adipocyte (Scale bar: 500μm); (E-H) the MSCs phenotypic molecular surface markers of hPDLSCs.*

Primary hPDLSCs migrated from periodontal ligament tissue explants and formed characteristic vortex-like colonies after 7 days of culture. Following purification by limited dilution, cells exhibited a homogeneous, spindle-shaped morphology under high magnification (Fig.S1 A-B). Multilineage differentiation potential was confirmed by ARS-stained mineralized nodules after osteogenic induction and Oil Red O-positive lipid droplets following adipogenic induction (Fig.S1 C-D). Flow cytometric demonstrated high expression of mesenchymal stem cell markers CD90 (99.6%), CD146 (99.5%), and STRO-1 (98.6%), with negligible expression of the hematopoietic marker CD34 (0.95%) (Fig.S1 E-H). These results indicate the successful isolation of hPDLSCs fulfill the minimal criteria for mesenchymal stem cells.
